# Basic Math in Monkeys and College Students

**DOI:** 10.1371/journal.pbio.0050328

**Published:** 2007-12-18

**Authors:** Jessica F Cantlon, Elizabeth M Brannon

**Affiliations:** Department of Psychology and Neuroscience, Center for Cognitive Neuroscience, Duke University, Durham, North Carolina, United States of America; Service Hospitalier Frederic Joliot, France

## Abstract

Adult humans possess a sophisticated repertoire of mathematical faculties. Many of these capacities are rooted in symbolic language and are therefore unlikely to be shared with nonhuman animals. However, a subset of these skills is shared with other animals, and this set is considered a cognitive vestige of our common evolutionary history. Current evidence indicates that humans and nonhuman animals share a core set of abilities for representing and comparing approximate numerosities nonverbally; however, it remains unclear whether nonhuman animals can perform approximate mental arithmetic. Here we show that monkeys can mentally add the numerical values of two sets of objects and choose a visual array that roughly corresponds to the arithmetic sum of these two sets. Furthermore, monkeys' performance during these calculations adheres to the same pattern as humans tested on the same nonverbal addition task. Our data demonstrate that nonverbal arithmetic is not unique to humans but is instead part of an evolutionarily primitive system for mathematical thinking shared by monkeys.

## Author Summary

Adult humans possess mathematical abilities that are unmatched by any other member of the animal kingdom. Yet, there is increasing evidence that the ability to enumerate sets of objects nonverbally is a capacity that humans share with other animal species. That is, like humans, nonhuman animals possess the ability to estimate and compare numerical values nonverbally. We asked whether humans and nonhuman animals also share a capacity for nonverbal arithmetic. We tested monkeys and college students on a nonverbal arithmetic task in which they had to add the numerical values of two sets of dots together and choose a stimulus from two options that reflected the arithmetic sum of the two sets. Our results indicate that monkeys perform approximate mental addition in a manner that is remarkably similar to the performance of the college students. These findings support the argument that humans and nonhuman primates share a cognitive system for nonverbal arithmetic, which likely reflects an evolutionary link in their cognitive abilities.

## Abstract 3

Monkeys have an ability to represent numerical values even though they lack linguistic abilities. The authors show that monkeys can also perform addition on numerical values and that they perform similarly to college students who are asked to add without counting.

## Introduction

The fact that humans and nonhuman animals represent numerical values nonverbally using a common cognitive process is well established [1–7]. Both human and nonhuman animals can nonverbally estimate the numerical values of arrays of dots or sequences of tones [8–12] and determine which of two sets is numerically larger or smaller [13–19]. When adult humans and nonhuman animals make approximate numerical comparisons, their performance is similarly constrained by the ratio between numerical values (i.e., Weber's law; [7]). Thus, discrete symbols such as number words and Arabic numerals are not the only route to numerical concepts; both human and nonhuman animals can represent number approximately, in a nonverbal code.

The parallel psychophysics for number discrimination in adult humans and various nonhuman animal species implicates an evolutionarily ancient system for representing number. Within this system, numerical representations take on an analog-magnitude format: mental representations of numerical values are proportional to the numerosities they represent (e.g., [8,16]). A key advantage for representing number in an analog format is that these representations can enter into arithmetic operations such as ordering and addition [7]. However, although there is a great deal of evidence that animals represent the ordinal relationships among numerosities (e.g., [14–17]), few studies have addressed whether animals can perform other arithmetic operations, and even fewer studies have directly compared performance between adult humans and nonhuman animals on the same arithmetic task.

Arithmetic operations—such as addition, subtraction, division, and multiplication—require mental transformations over numerical values. Addition is an arithmetic operation that involves combining two or more quantitative representations (addends) to form a new representation (the sum). The ability to mentally combine representations is inherent to many aspects of human cognition including language and symbolic mathematical expression [20]. One possibility, then, is that the ability to combine representations, whether linguistic or arithmetic, is unique to humans.

There is, however, already some evidence that nonhuman animals can perform approximate, nonverbal addition on numerical values [21–28]. For instance, Flombaum, Junge, and Hauser [21] found that when untrained rhesus monkeys watched as two groups of four lemons were placed behind a screen, they looked longer when the screen was lowered to reveal only four lemons (incorrect outcome) than when the correct outcome of eight lemons was revealed (see also [22,23]). Thus, as measured by their looking time, monkeys spontaneously form numerical expectations when they view addition events. Moreover, Beran and colleagues [24–26] have demonstrated that nonhuman primates reliably choose the larger of two food quantities, even when this requires tracking one-by-one additions to multiple caches over time. Such data provide important evidence that animals can form numerical representations when this requires one-by-one accumulation, but they leave open the question of whether animals can perform nonverbal arithmetic by combining set-level representations.

Other studies have trained animals to associate arbitrary symbols with numerosities and then tested the animals' ability to add symbols [27,28]. For example, pigeons reliably chose the combination of two symbols that indicated the larger amount of food [27]. However, when the number of food items associated with the symbols was varied but total reward value (mass) was held constant, the pigeons failed to determine the numerical sum of the food items, suggesting that they performed the addition task by representing the total reward value represented by the two symbols, rather than by performing numerical arithmetic. Thus, food items may not be an optimal stimulus for testing pure numerical arithmetic in nonhuman animals.

To date, the most persuasive test of arithmetic in a nonhuman animal was conducted on a single chimpanzee [28]. In this study, a symbol-trained chimpanzee chose the Arabic numeral that corresponded to the sum of hidden sets of oranges, for sets that summed to less than four items, over 14 test trials. In contrast, studies of adult human nonverbal addition have tested many trials with a large range of numerical values and arithmetic problems (e.g., [4,13,29,30]). Thus, although there is suggestive prior evidence that nonhuman animals may perform mental arithmetic, the data are not definitive. An important limitation of all prior studies of nonhuman arithmetic is that they used drastically different methods from those used to test adult human nonverbal arithmetic. The degree to which nonhuman arithmetic parallels the nonverbal arithmetic of adult humans is therefore undetermined.

Several studies provide compelling evidence that without verbally counting, adult humans can choose the approximate sum of two or more sets. These studies required subjects to add two arrays of arbitrary elements and then select the correct sum, over hundreds of trials, testing a wide range of numerical values (e.g., [4,13,29,30]). For example, in one study [13], adults were presented with two arrays of dots (of 1–62 elements) and were required to mentally add the numerical values of the sets to determine whether a third test array was approximately equal to their sum. Performance was modulated by the subjective difference between the correct sum and the test array (i.e., Weber's law); accuracy declined as the ratio between the choices (smaller value/larger value) approached one. Thus, adult humans have the capacity for precise symbol-based arithmetic, and they are also able to perform approximate addition on nonsymbolic quantities.

**Figure 1:**
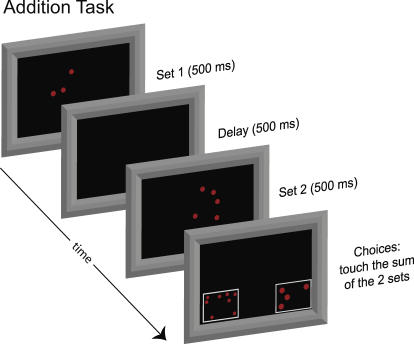
The Addition Task Monkeys and humans were presented with one set of dots (set 1), followed by a brief delay after which a second set of dots was presented (set 2). Then, two choices (the sum and the distractor) were presented, and monkeys were rewarded for touching the choice that represented the numerical sum of the two sets.

Our goal was to compare directly the nonverbal arithmetic abilities of monkeys and adult humans using the same task and stimuli. Monkeys (*n* = 2) and college students (*n* = 14) were presented with two sets of dots on a touch screen monitor separated by a delay (Figure 1). Following the presentation of these two sets, subjects were required to choose between two arrays: one with a number of dots equal to the sum of the two sets and a second, distractor array, which contained a different number of dots. Our results indicate that monkeys perform approximate mental addition in a manner comparable to college students tested on the same addition task.

## Results

### Addition Performance of Monkeys

**Figure 2:**
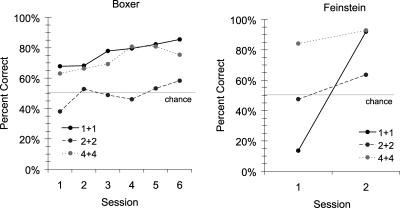
Monkeys' Acquisition of the Addition Task Each session was approximately 250 trials, divided equally among the three problem types (1 + 1, 2 + 2, and 4 + 4). Feinstein and Boxer required two and six sessions, respectively, to reach above-chance performance on all three trial types (binomial tests of accuracy versus chance: Boxer: 1 + 1, *n* = 62, 0.85 versus 0.5, *p* < 0.001; 2 + 2, *n* = 127, 0.58 versus 0.5, *p* < 0.05; 4 + 4, *n* = 61, 0.75 versus 0.5, *p* < 0.001. Feinstein: 1 + 1, *n* = 149, 0.92 versus 0.5, *p* < 0.001 ; 2 + 2, *n* = 184, 0.64 versus 0.5, *p* < 0.001; 4 + 4, *n* = 167, 0.93 versus 0.5, *p* < 0.001).

During the initial phase of training for the addition task, we presented monkeys with a limited set of addition problems (1 + 1 = 2, 4, or 8; 2 + 2 = 2, 4, or 8; 4 + 4 = 2, 4, or 8). Monkeys performed at a level significantly greater than chance on each of these three problems within 500 trials (Figure 2). Performance on the 2 + 2 addition problem was significantly worse than performance on the 1 + 1 and 4 + 4 problems for both monkeys (*p* < 0.05). This finding suggests that monkeys' performance resulted from approximate arithmetic even during this early stage of training, because the discrimination ratio of the sum to the choice stimuli was more difficult for the 2 + 2 problems than either the 1 + 1 or 4 + 4 problems. More specifically, the distractor values we tested resulted in more difficult numerical discriminations for the 2 + 2 problems (mean discrimination ratio = 0.5) than the 1 + 1 and 4 + 4 problems (mean discrimination ratios = 0.38). However, during this initial training, monkeys may have learned the specific relationships between the addends and sums for this limited set of problems rather than performing true addition. That is, monkeys may have formed associations between a particular pair of addends and its resulting sum.

**Figure 3:**
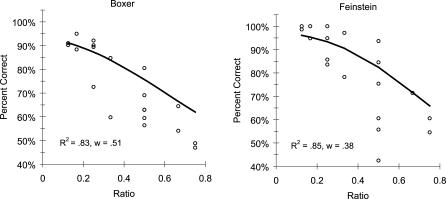
Monkeys' Addition Performance Was Ratio-Dependent Both monkeys' accuracy on each of the addition problems was modulated by the numerical ratio between the correct sum and the distractor choice. Solid lines show the predicted data from Equation 1. *R* ^2^ values reflect the fit between the predicted and actual data. *w* represents the precision with which monkeys selected the correct sum from the distractor choice based on the best fitting predicted data. As the ratio (small/large) between the sum and the distractor approached one, accuracy declined. Chance = 50%.

Next, we expanded the range of addition problems to include the numerical values 2, 4, 8, 12, and 16. All possible permutations of addends summing to these values were tested (e.g., sum of 8 = 1 + 7, 2 + 6, 3 + 5, 4 + 4, 5 + 3, etc.) and all values were equally likely to occur as correct and incorrect choices. Monkeys' performance was modulated by the ratio between the numerical values of the choice stimuli; they performed significantly better when the numerical difference between the choice stimuli was easier to discriminate (Figure 3).

To confirm that monkeys' performance was modulated by the ratio between the numerical values of the sum and choice stimuli, we tested monkeys' performance against a mathematical model developed by Stanislas Dehaene ([4,13] and see [31] for full description of model] for human nonsymbolic arithmetic performance.

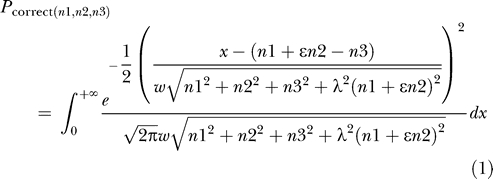



This model represents each of the two addends (*n*1*, n*2) and the distractor value (*n*3) as a Gaussian distribution with a mean equal to their numerical value and a standard deviation that increases proportional to the mean. In addition, the model includes a parameter for the internal Weber fraction (*w*), which reflects the amount of variability, or noise, in the distributions. This version of the model includes a parameter (λ), which modulates additional variability associated with the sum of the addends after they have been added together and stored, temporarily, in memory [31]. Here, the best fitting value for λ ranged from 1.3–1.5, although simpler implementations of this model have set λ = 0 (e.g., [4,13]). As a consequence of these parameters, the predicted probability of selecting the correct sum from the distractor choice depends on the ratio between the numerical values of the sum and distractor choice, the degree to which numerical values at this ratio are internally distinct (*w*), and the added variability associated with forming the initial representation of the sum (λ). In short, this model predicts the probability of success on a given addition problem under Weber's law.

We implemented this model to obtain the predicted performance for the addition problems tested and used a goodness of fit test (*r* ^2^) to determine the *w* that best accounted for the monkeys' performance (Figure 3). We found that this model predicted a significant amount of the variance in monkeys' performance (Boxer: *R* ^2^ = 0.83, *p* < 0.0001; Feinstein: *R* ^2^ = 0.85, *p* < 0.0001), demonstrating that monkeys' addition performance was modulated by the numerical ratio of the sum and distractor. It is noteworthy that even during this training period, monkeys' approach to these addition problems was comparable to the process used by adult humans on parallel tasks (e.g., [4,13]).

**Figure 4:**
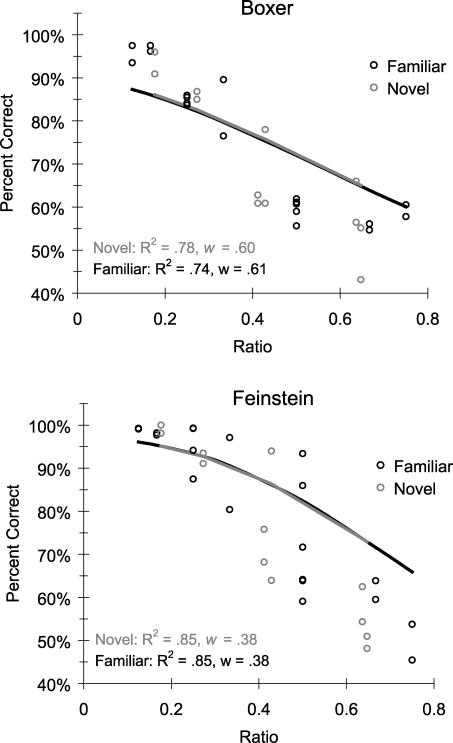
Monkeys Can Solve Novel Addition Problems Both monkeys performed significantly above chance on the novel and familiar addition problems, and their performance was similarly modulated by the ratio between the numerical values of the sum and distractor choices. Solid lines represent the predicted data from Equation 1 at the best-fitting *w*. *R* ^2^ values reflect the fit between the predicted and actual data for familiar trials (black) and novel trials (gray; all *p*'s < 0.0001).

To determine whether monkeys relied on an abstract mental addition process that could be applied to both familiar and unfamiliar numerical values, we tested them with novel addition problems. We tested all possible addends of the novel sums 3, 7, 11, and 17. To prevent learning on these novel test trials, monkeys were rewarded regardless of which of the two choice stimuli they selected as the sum. Performance on these nondifferentially reinforced test trials was significantly greater than that predicted by chance (one-sample *t*-test of accuracy on novel addition problems vs. chance; Boxer: 70% versus 50%; *t*(11) = 4.20, *p* < 0.01; Feinstein: 75% versus 50%; *t*(11) = 4.45, *p* < 0.001). Furthermore, addition performance with novel numerical values was modulated by numerical ratio, just as with the familiar values. That is, performance on both the familiar and novel numerical values decreased as the ratio between the choice stimuli approached one; the model of ratio-dependent addition performance presented in Equation 1 well-accounted for monkeys' performance on both trial types (Figure 4). This finding demonstrates that monkeys performed addition on the novel numerical values using the same cognitive process that they used for the familiar numerical values. Therefore, monkeys added the values of the two sets of elements together regardless of the absolute value of the sets and independent of their familiarity with particular values or addition problems.

Additional analyses confirmed that monkeys' performance was based purely on the sum of the two addends. First, monkeys were not simply choosing the numerically larger of the two choice stimuli. Monkeys performed significantly above chance on addition problems regardless of whether the distractor stimulus was larger or smaller than the sum (distractor larger: Boxer, 75%, *t*(15) = 6.43, *p* < 0.001, Feinstein, 82%, *t*(15) = 6.32, *p* < 0.001; distractor smaller: Boxer, 70%, *t*(15) = 4.69, *p* < 0.001, Feinstein, 75%, *t*(15) = 5.85, *p* < 0.001). In addition, both monkeys performed significantly better than chance, even when the first addend was equal to the numerical value of the distractor choice (binomial tests; Boxer, *n* = 178, 0.75 versus 0.5, *p* < 0.001; Feinstein, *n* = 149, 0.81 versus 0.5, *p* < 0.001). Similarly, accuracy was better than chance when the distractor value was equal to the second addend (Boxer, *n* = 148, 0.76 versus 0.5, *p* < 0.001; Feinstein, *n* = 143, 0.73 versus 0.5, *p* < 0.001) and the largest addend (Boxer, *n* = 114, 0.62 versus 0.5, *p* < 0.01; Feinstein, *n* = 98, 0.62 versus 0.5, *p* < 0.01). Thus, performance was unimpaired when a strategy based on matching a single addend predicted the incorrect choice. Rather than using a simple heuristic, monkeys mentally added the two sets and based their choices on the sum of the two addends.

To confirm that monkeys were not performing addition across the spatial extent of the dots as opposed to their number, we examined their performance as a function of the cumulative surface area of the addends and choice stimuli. As described in Materials and Methods, the cumulative surface area of the elements in the stimuli was varied to create trials in which a strategy based on the cumulative surface area of the elements would result in error. On approximately 25% of all trials, the cumulative surface area of the dots in the distractor stimulus was closer to the cumulative surface area of the dots in the two addends. If monkeys were using the cumulative surface area of the addition sets to perform this task, their performance should be below chance on these trials, because the incorrect numerical choice was the correct choice for cumulative surface area. This was not the case. Instead, monkeys performed significantly above chance on this subset of trials, indicating that they based their choices on the numerical sum of the objects, not their surface area (binomial test of accuracy on area control trials versus chance; Boxer: *n* = 1571, 0.83 versus 0.5, *p* < 0.00001; Feinstein: *n* = 1460, 0.88 versus 0.5, *p* < 0.00001).

Finally, performance was equivalent on trials that required addition and “single-set” trials that did not require addition (dependent sample *t*-test on addition trials versus single-set trials; Boxer: *t*(31) = 0.56, *p* = 0.58; Feinstein: *t*(31) = −0.70, *p* = 0.49). On single-set trials, all of the dots were presented simultaneously, in a single set, and monkeys simply had to select the correct numerical match between this single set and one of the two choice stimuli (see [13] for a similar result with adult human subjects). The numerical values tested on these trials were identical to those tested on the addition trials. The equivalent, ratio-dependent performance on addition and single-set trials confirms that monkeys used a mental computation during addition that is linked to their broader set of numerical skills in the sense that they invoke a common form of numerical representation during addition and numerical estimation

### Comparison of Addition Performance of Monkeys and Adult Humans

Overall accuracy across the 40 different addition problems was higher for adult humans (mean = 94%) than for monkeys (mean = 76%) on the addition trials (*t*(38) = 3.90, *p* < 0.001). However, the mean response time of monkeys (mean = 1,099 ms) and humans (mean = 940 ms) was not significantly different (*t*(38) = 1.43, *p* = 0.16). Thus, humans responded at the same rate as monkeys but were more accurate overall.

**Figure 5:**
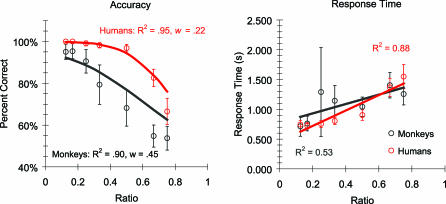
Monkeys Perform Addition like Humans Monkeys and humans exhibited ratio-dependent accuracy and response time when solving addition problems. For accuracy (left panel), solid lines show the predicted data from Equation 1 for humans (red) and monkeys (gray) at the best fitting *w*. The *R* ^2^ values for accuracy show the strength of the fit. Response times (right panel) are fit with a linear function, and the corresponding *R* ^2^ values are reported. Error bars reflect the standard error among subjects.

Despite these quantitative differences in performance, however, monkeys and humans produced qualitatively similar patterns of accuracy and response time in the addition task (Figure 5). Monkeys and humans alike exhibited a correlation between the numerical ratio of the choice stimuli and their speed in choosing the correct arithmetic outcome (humans: *R* ^2^ = 0.88, *p* < 0.005; monkeys: *R* ^2^ = 0.53, *p* = 0.06). Moreover, predicted performance from the model of ratio-dependent addition captured the accuracy data from both monkeys and humans (humans: *R* ^2^ = 0.95, *p* < 0.0001; monkeys: *R* ^2^ = 0.90, *p* < 0.0001). The precision variable in the model (*w*) that produced the best-fitting predicted performance for humans (*w* = 0.22) indicated that humans were able to make finer numerical discriminations than monkeys were (*w* = 0.45). Overall, however, the robust relationships among numerical ratio, accuracy, and response time indicate that the primary constraint for humans and monkeys in solving addition problems was the numerical ratio between the correct sum and the distractor choice. The data from the individual numerical values of the sum–distractor pairs that contributed to this analysis are presented in Figure S1.

**Figure 6:**
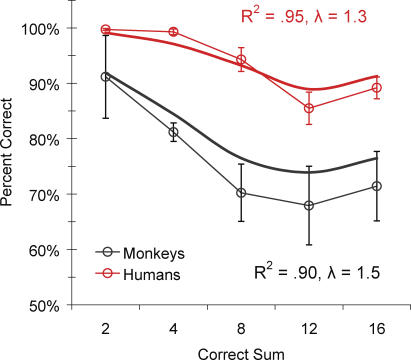
The Effect of the Magnitude of the Sum on Accuracy for Addition Trials Monkeys and humans performed less accurately as the numerical magnitude of the sum of the sample sets increased. Bolded lines show the predicted data from Figure 5 presented as a function of sum size. Error bars reflect the standard error among subjects.

In addition to the effect of numerical ratio, there was also an effect of the numerical magnitude of the sum on monkeys' and humans' performance; accuracy decreased as the sum increased for the addition trials (Figure 6). This sum size effect is also predicted by Equation 1, because it includes a parameter (λ) that modulates additional variance contributed by the representation of the sum, after the addends have been added together and stored in memory [31]. The effect of sum size in our data confirms that the numerical magnitude of the sum of the two sets contributes additional noise to the representational process of adding two sets together and choosing the correct sum from two choice stimuli.

Finally, like monkeys, adult humans performed similarly on addition trials and single-set trials. Humans exhibited no significant difference in accuracy between addition trials and single-set trials in which all of the elements were presented all at once (*t*(13) = 1.19, *p* = 0.26). The lack of a difference in performance between comparison and addition has also been found when adult humans perform approximate arithmetic [13]. Thus, humans' capacity to perform rapid, approximate arithmetic appears to be linked to their broader skill set for estimating numerical values [16]. However, one peculiar finding is that humans' performance on these single-set trials was also affected by the magnitude of the sample stimulus above and beyond the effect of numerical ratio. Specifically, adult humans were more accurate at a given ratio when the sample value was relatively small on single-set trials (*r* = −0.48, *p* < 0.05) although this was not the case for monkeys (*r* = −0.17, *p* = 0.5).

Overall, the data from the addition performance of adult humans reinforce the claim that the basic arithmetic ability we have observed in the current study belongs to a primitive mathematical toolkit that deals in approximate, analog representations of numerical values with a limiting performance factor of numerical ratio. Monkeys invoke this mathematical system to solve quantitative problems, whereas humans invoke this primitive system when precise, symbolic mathematics is not a viable option, as was the case in the current study.

## Discussion

Our results provide definitive evidence that monkeys can perform mental addition. Furthermore, monkeys' accuracy in combining numerical values was ratio-dependent, suggesting that they performed addition by combining analog-numerical representations. The qualitative similarity between the performance of monkeys and humans in this study is striking; when monkeys and humans nonverbally add two sets of objects together to represent their sum, their performance is similarly modulated by the ratio between the numerical values of choice stimuli (see also [4]). Humans and nonhuman primates thus appear to share a cognitive system for basic nonverbal arithmetic, which likely reflects an evolutionary link in their cognitive abilities.

Although it is impossible to know precisely the function for which numerical arithmetic may have been selected in our evolutionary past, a few studies have shown that extant nonhuman animals use numerical information to determine the number of animals in an unfamiliar group during territorial disputes [32,33] and to choose a relatively large amount of food during foraging [34]. Numerical information thus appears to be influential in both social and foraging decisions. Numerical arithmetic may be important during social and foraging decisions under circumstances in which groups of animals or food items are widely separated in space and/or time. For example, if conspecifics or food items are widely distributed in space or time, an animal may have no choice but to perform addition in order to update an initial quantitative representation.

Our results demonstrate that when monkeys mentally add numerical values together, their performance is modulated by numerical ratio, just as when they compare or equate stimuli based on numerical values [16]. Thus for monkeys, addition is a computation that belongs to a mathematical toolkit with an overarching set of psychological principles. In support of this claim, recent studies have demonstrated that nonhuman primates exhibit ratio-dependent performance when they abstract numerical values across stimuli with high perceptual variability [17] and even across sensory modalities (Jordan KE, MacLean EL, Brannon EM, unpublished data). Additionally, the process that monkeys and humans use to compare numerical values seems to obey the same algorithm [15,16]. It is becoming increasingly apparent that the set of nonverbal mathematical skills shared by humans and nonhuman animals is remarkably abstract and computationally powerful.

The precise computational and neural mechanism by which humans or monkeys perform nonverbal addition is unknown. Gelman and Gallistel [7] proposed that nonverbal addition functions in a manner parallel to histogram arithmetic. Discrete quantities are represented as analog magnitudes that are isomorphic to the quantities they represent, much like the process by which analog machines represent discrete quantities in currents or voltages. In this sense, mental representations of numerical values are analogous to the bars on a histogram in which height is an index of numerical magnitude. To perform addition, these analog representations of number might be combined in a manner equivalent to spatially combining the bars of a histogram. In this case, the bars of the histogram represent the numerical sum of sets of discrete objects. The result of histogram addition is a new mental magnitude (the sum) that is directly proportional to the combined numerical magnitude of the two original quantities. This kind of mechanism may underlie nonverbal numerical arithmetic in humans and nonhuman animals.

In the current study, when monkeys selected the sum of the two sets of dots, they based their decisions on a representation (the sum) that they generated by mentally combining two existing numerical representations (the addends). The ability to combine mental representations is a capacity that humans invoke regularly to solve cognitive problems and especially to produce symbolic mathematical expressions. Our results demonstrate that, like humans, monkeys are capable of combining mental representations of numerical values together to solve mathematical problems. Indeed, the qualitative similarity between the performance of monkeys and humans on our addition task is evidence that they likely compute simple nonverbal arithmetic outcomes in much the same way. This conclusion is bolstered by our finding that a single equation accounts equally well for monkeys' and humans' success in performing addition nonverbally (see also [31]).

Studies of animal cognition from a variety of domains describe cases in which animals appear to perform computations that require combining mental representations. For example, to locate objects via echolocation, bats must combine information from phase shifts in both the original and reflected sound emissions across many different frequencies [6,35]. In addition, rats can integrate information about the metric relations among surfaces in their environment with information about their position within that space during navigation (e.g., [36]). Our data add to the evidence that nonhuman animals combine representations by providing evidence of combinatorial computations that operate over numerical representations of discrete objects to represent approximate arithmetic outcomes.

In short, our data advance the hypothesis that numerical addition is a component of the primitive, language-independent set of numerical capacities that has a common evolutionary origin among primates, including humans. More broadly, our data demonstrate that the ability to combine mental representations, which is a characteristic of sophisticated aspects of human cognition, is a capacity that nonhuman animals use within the numerical domain. These findings underscore the existence of extraordinary continuity in the processes governing numerical thought for human and nonhuman primates.

## Materials and Methods

### Subjects.

Nonhuman primate subjects were two adult female rhesus macaques, named Feinstein and Boxer, who were socially housed along with two other rhesus macaque females. All animal care procedures are in accordance with an IACUC protocol. Human participants were 14 adults (mean age = 23 y, standard deviation = 3.45, 5 male) that currently attend Duke University.

### Task.

Monkeys were tested in sound-attenuated touch screen booths while seated in Plexiglas primate chairs. Adult humans were tested at a touch screen computer station. For both species, stimuli were presented on a touch screen in randomly selected locations. To begin a trial, subjects were required to press a start stimulus, a small red square presented in the bottom left corner of the screen. Following this response, two sets of dots were presented, separated by a delay of 500 ms. Then, subjects were presented with two choice stimuli and were required to select the stimulus that contained the numerical sum of these two sample sets. A trial terminated when a subject touched one of the two choice stimuli.

Both species received positive visual (light-up border) and auditory (chime) feedback for correct choices and negative visual (black screen) and auditory (warning tone) feedback for incorrect responses. Incorrect responses were also followed by a 2–5 s timeout period. Monkeys were also rewarded with small amounts of Kool-Aid for selecting the correct sum. When monkeys failed to select the correct choice, they received no juice reward. Humans were given $10 to participate in the study.

For all subjects, all numerical values tested were equally likely to occur as the correct and incorrect choices; thus the incorrect choice could be smaller or larger than the sum on any trial. Stimuli were trial-unique, in the sense that a computer program randomly selected the sizes and locations of the elements in each array from a parameter distribution. Thus the sizes and locations of the elements could not be used to solve the task. A video of a monkey and an adult human performing this task can be found at: http://rd.plos.org/pbio.0050328.

### Training monkeys.

Prior to training on the addition task, monkeys were trained on a numerical matching task in which a sample array of 1–9 dots was presented and they were rewarded for selecting the array that numerically matched the sample set from two choices (see [9]). Monkeys reached a 70% criterion on this numerical matching task before training on the addition task.

For the initial training on the addition task, monkeys were presented with a limited range of addition problems: 1 + 1 = 2, 4, or 8; 2 + 2 = 2, 4, or 8; 4 + 4 = 2, 4, or 8. Monkeys completed ∼9,000 trials on this phase of training; however, as reported in the results section, their performance was above chance within the first 500 trials. Next, we expanded the range of addition problems by testing all possible addends of the sums 2, 4, 8, 12, and 16. For example, when 8 was the sum, the addends could be 1 + 7, 2 + 6, 3 + 5, 4 + 4, 5 + 3, 6 + 2, or 7 + 1. Each sum was equally likely to occur as the correct and incorrect choices. Monkeys completed approximately 5,000 trials on this phase of training before we tested them with novel addition problems. Throughout training and testing, we included trials in which the monkeys were not required to add. On these trials, a single set of dots was presented on monkeys were required to select the choice stimulus that corresponded to its numerical value. These single-set trials were analyzed separately from the addition trials as a measure of monkey's numerical performance in the absence of arithmetic computation.

### Testing monkeys.

Monkeys were tested on addition problems that they have never been trained to compute. All possible addends of the novel values 3, 7, 11, and 17 were tested. These novel values were equally likely to occur as correct and incorrect choices during test trials. Thus, there were 12 different novel sum-distractor pairs. Approximately 50 trials were completed by each monkey on each novel pair. Novel addition problems were presented randomly within a session and comprised 20% of the total trials. To prevent monkeys from learning the solutions to the novel problems, they were rewarded no matter which of the two choice stimuli they selected as the sum. During the second half of testing sessions, a green rectangle appeared during the delay between the two addend sets rather than a blank black screen.

### Task instructions for adult humans.

Adult humans were instructed to press the start stimulus to initiate each trial and then to attend to the number of dots in each set, add them together without verbally counting, and rapidly select the box that contained their sum from two choices. The task was demonstrated by the experimenter for 3–5 trials, the subject practiced the task for 3–5 trials, and then testing began.

### Testing adult humans.

The task used to test adult humans was identical to that used for the final phase of monkey training. Adult humans were tested on all possible addends of the sums 2, 4, 8, 12, and 16. Each sum was equally likely to occur as the correct and incorrect choices. Half of the trials were single-set trials, wherein the total number of items was presented simultaneously in a single set rather than across two sets. Each adult completed 500 trials on this task over a 50-min period.

### Stimuli.

For both species, the training and testing stimuli consisted of red dots on a black background. Stimuli were trial-unique in the sense that the surface area, location, and spacing of the elements were varied. For the sample sets, the location of each element in a set was randomly drawn (barring overlap among elements) from all *x*- and *y*- coordinates within approximately 10 cm in any direction of the center of the screen. For each choice stimulus, elements were randomly placed (again, barring overlap among elements) in a 9 cm × 7.5 cm stimulus. To control for cumulative surface area, the physical sizes of the elements were varied such that the cumulative surface area of the sample sets varied between 250 and 11,000 pixels, and the cumulative surface area of the choice stimuli was either 1,000 or 10,000 pixels. For each of the numerical values tested, the cumulative surface area values of the choice stimuli were equally likely to occur as the correct and incorrect choice. Consequently, on a percentage of trials, the incorrect choice had the closer cumulative surface area value to the cumulative surface area of the sample sets. If monkeys were using cumulative surface area to perform this task, as opposed to number, they would have failed to choose the correct sum on these trials. In the Results section, we separately analyzed trials in which a strategy based on the cumulative surface area of the elements would lead to failure.

## Supporting Information

Figure S1 Accuracy on Addition Trials for Monkeys and Adult Humans on Individual Sum–Distractor Pairs Each sum represents all possible combinations of addends that result in that sum. For example, a sum of 8 consisted of problems 1 + 7, 2 + 6, 3 + 5, 4 + 4, 5 + 3, 6 + 2, and 7 + 1. Chance is 50% on this task. Error bars reflect variability among subjects.
(916 KB EPS)
